# Rare and Aggressive Disease: Urinary Bladder Leiomyosarcoma

**DOI:** 10.3390/jcm14175999

**Published:** 2025-08-25

**Authors:** Zilvinas Venclovas, Kotryna Simkunaite, Vaidas Pijadin, Stasys Auskalnis, Mindaugas Jievaltas, Tomas Navickis, Daimantas Milonas

**Affiliations:** 1 Department of Urology, Lithuanian University of Health Sciences, LT-44307 Kaunas, Lithuania; stasys.auskalnis@lsmu.lt (S.A.); mindaugas.jievaltas@kaunoklinikos.lt (M.J.); daimantas.milonas@lsmu.lt (D.M.); 2 Faculty of Medicine, Medical Academy, Lithuanian University of Health Sciences, LT-44307 Kaunas, Lithuania; kotryna.simkunaite@stud.lsmu.lt (K.S.); vaidas.pijadin@stud.lsmu.lt (V.P.); 3 Department of Pathological Anatomy, Lithuanian University of Health Sciences, LT-44307 Kaunas, Lithuania; tomas.navickis@lsmu.lt

**Keywords:** leiomyosarcoma, nonurothelial bladder tumor, cystectomy, rare disease, oncology

## Abstract

**Background**: Bladder leiomyosarcoma is an extremely rare non-urothelial malignancy, accounting for less than 0.1% of all bladder tumors. It presents significant diagnostic and therapeutic challenges due to its aggressive nature and the absence of standardized treatment protocols. **Case presentation**: We report the case of a 61-year-old woman who presented with hematuria, dysuria, and suprapubic pain. Imaging revealed a large, locally invasive bladder mass, and histopathological examination following transurethral resection confirmed leiomyosarcoma. The patient underwent radical cystectomy with resection of adjacent bowel segments and urinary diversion. Histology showed a high-grade leiomyosarcoma (pT3N0) with extensive necrosis and a high mitotic index. Two months postoperatively, peritoneal dissemination was detected. Systemic chemotherapy with dacarbazine and doxorubicin initially led to the regression of metastases, but disease progression occurred within months, including lung, liver, and bone metastases. Palliative radiotherapy and second-line chemotherapy were initiated. As of now, 16 months have elapsed since surgery. **Conclusions**: This case underscores the aggressive clinical course of bladder leiomyosarcoma despite multimodal therapy and the urgent need for individualized management strategies. Given its rarity, this case contributes to the limited literature and highlights the importance of vigilant follow-ups and further studies to establish evidence-based treatment protocols.

## 1. Introduction

Bladder cancer (BCa) was the ninth most commonly diagnosed cancer and the thirteenth leading cause of cancer death worldwide in 2022 [1]. Urothelial carcinoma is the most prevalent subtype, accounting for over 90% of all bladder cancer cases [2]. Non-epithelial bladder tumors comprise less than 5% of all bladder malignancies, with leiomyosarcoma representing approximately 1% of primary bladder tumors [3,4]. This rare tumor originates from smooth muscle tissue and primarily affects adults [5] and its aggressive nature complicates early diagnosis and management [6]. Clinical symptoms such as hematuria, dysuria, suprapubic pain, and nocturia are common but nonspecific, often mimicking other malignant or benign urological conditions [7]. Diagnosis is confirmed through histopathological examination, which is essential to differentiate leiomyosarcoma from histologically similar entities such as leiomyomas or inflammatory myofibroblastic tumors [8]. Histologically, leiomyosarcomas exhibit spindle-shaped cells with pronounced atypia, increased mitotic activity, and occasionally necrotic areas [9].

Multimodal therapy–comprising surgery, chemotherapy, and radiation–has been proposed to improve survival; however, evidence is limited due to the tumor’s rarity, frequent presentation with metastasis, uncertain etiology, and lack of standardized treatment guidelines [9]. Surgery, particularly radical cystectomy, remains the cornerstone of management. Nonetheless, despite aggressive therapy, the tumor carries a high risk of local recurrence and distant metastasis [3]. The median survival after radical surgery is approximately several months, while the untreated survival is usually less than six months [10,11]. This report presents a case of bladder leiomyosarcoma with subsequent metastatic progression and initiation of palliative therapy.

## 2. Case Report

A 61-year-old non-smoking woman was presented to the emergency department with an urgent need to urinate, pain, and a burning sensation during urination. She also reported a three-month history of hematuria, dysuria, and suprapubic pain. Her past medical history included a hysterectomy with pelvic lymphadenectomy for multiple uterine myomas five years prior. Malignancy was not confirmed.

**Figure 1:**
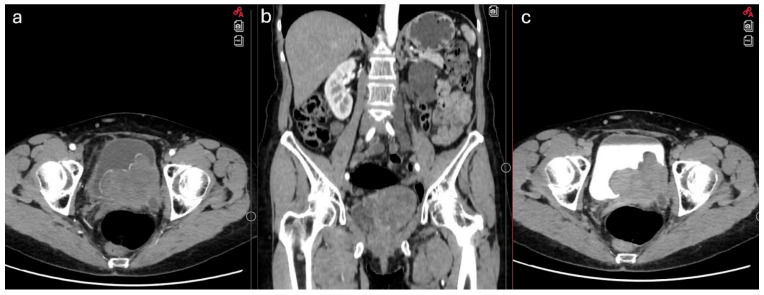
Preoperative CT showing a bladder mass. (**a**) axial view in the arterial phase; (**b**) coronal view; (**c**) axial view in the delayed phase.

Ultrasound revealed a seven cm bladder mass. Computer tomography of chest abdomen, and pelvis (CT CAP) and pelvic MRI tests were performed. They demonstrated a 7.5 × 6.2 × 7.3 cm polycystic, contrast-enhancing bladder tumor infiltrating the left ureter, perivesical fat, vagina, and possibly the sigmoid colon (Figure 1). No pathological lymphadenopathy was observed. The left kidney showed hydronephrosis. Dynamic renal scintigraphy confirmed that the left kidney was non-functional. Blood tests were within normal limits.

Transurethral resection of the bladder tumor (TURBT) was performed. The tumor extensively involved the entire left bladder wall, and due to its size, complete radical resection was not feasible. The right ureteral orifice was patent; however, the left ureteral orifice was not visualized, prompting the placement of a left nephrostomy. Histopathological examination confirmed leiomyosarcoma.

One month after the primary TURBT, open radical cystectomy with pelvic lymphadenectomy was performed. A segment of the small intestine adherent to the tumor was resected. Additionally, a portion of the sigmoid colon adherent to the tumor was resected and a colostomy was created. A right ureterocutaneostomy was performed, while the nonfunctional left kidney was preserved owing to prolonged operative time, significant blood loss, and extensive right ureteral adhesions.

**Figure 2:**
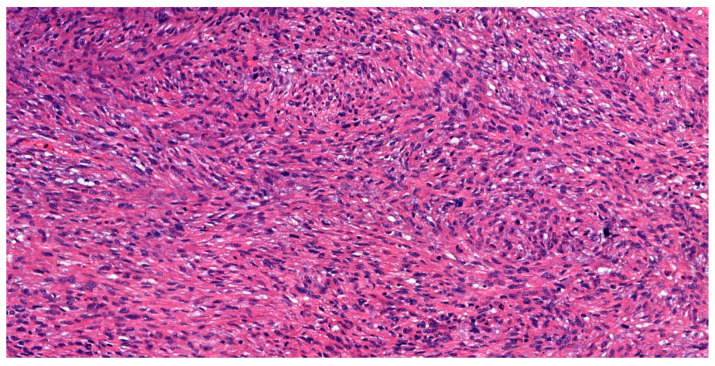
High magnification showing increased mitotic activity in the spindle-shaped tumor cells (H&E, ×400).

**Figure 3:**
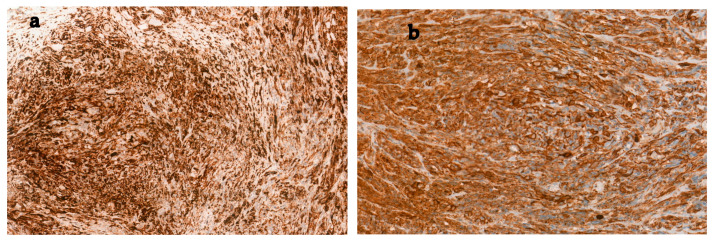
Immunostaining of the tumor for: (**a**) actin (positive); (**b**) caldesmon (positive).

Histopathology of the cystectomy specimen confirmed pT3 N0, grade G2 leiomyosarcoma composed of medium to large spindle-shaped cells arranged in fascicular patterns with polymorphic nuclei (Figure 2). The mitotic index reached up to 18 per 10 HPF. Necrosis was more extensive, involving up to 50% of the tumor area. The tumor tissue infiltrated the wall of the large intestine. Although the adjacent ureter was adherent to the bladder wall, no evidence of neoplastic infiltration was observed. As in previous studies of the TURBT specimen, immunohistochemically the tumor cells stained positively for smooth muscle actin and caldesmon, but negatively for desmin, pan-cytokeratin (panCK), CD31, CD34, and S100P markers (Figure 3).

Two months later, CT CAP scan revealed peritoneal dissemination. The patient received six cycles of dacarbazine–doxorubicin chemotherapy, resulting in the regression of metastases. The patient remained in remission for five months. CT scans were performed every three months; however, the most recent CT scan, performed thirteen months after the surgery, demonstrated numerous small metastatic lesions (up to 0.8 cm in size) in both lungs. The liver appeared to be of normal size; however, a hypovascular metastatic lesion measuring approximately 3.2 × 2.9 cm was identified on the medial surface of segments S5/S6. A pathological lymph node approximately 1.1 cm in size was observed in the mesentery of the small intestine and the pelvic region. An MRI of the pelvis revealed a metastatic lesion in the body of the L5 vertebra. Palliative radiotherapy (20 Gy in 5 fractions) was initiated for the L5 metastasis, along with systemic chemotherapy consisting of up to six cycles of gemcitabine and docetaxel. At present, 16 months have passed since the surgery.

## 3. Discussion

Bladder leiomyosarcoma is a rare, non-urothelial malignancy, comprising less than 0.1% of all non-epithelial tumors [12]. It most commonly affects individuals in their fifth to seventh decades of life and has a slight male predominance [13,14]. The clinical presentation, which typically includes hematuria, dysuria, and suprapubic discomfort, is nonspecific and may overlap with more common conditions such as urothelial carcinoma or benign inflammatory bladder diseases. In our case, the patient’s persistent symptoms and hematuria prompted further imaging and histopathological evaluation.

Diagnostic workup generally begins with imaging modalities such as ultrasound and CT, which are valuable in determining tumor size, local invasion, and adjacent organ involvement. In the context of diagnostic imaging, current guidelines for upper tract urothelial carcinomas emphasize contrast-enhanced CT urography as a first-line tool due to its high pooled sensitivity and specificity (92% and 95%, respectively), while MRI remains a secondary option when contrast is contraindicated [15]. However, imaging alone cannot reliably distinguish mesenchymal tumors. Therefore, histopathology and immunohistochemistry (IHC) are crucial for diagnosis, as demonstrated in our case following TURBT. Histologically, bladder leiomyosarcoma presents as an infiltrative neoplasm composed of intersecting fascicles of spindle cells with varying degrees of nuclear atypia, mitotic figures, and sometimes necrosis. IHC is instrumental in confirming the diagnosis and excluding other spindle cell neoplasms. Leiomyosarcomas are typically positive for smooth muscle actin (SMA), desmin, and h-caldesmon, and negative for epithelial markers like cytokeratin (CK) or epithelial membrane antigen (EMA), helping to distinguish them from sarcomatoid urothelial carcinoma or inflammatory myofibroblastic tumors [7,16].

Due to the rarity of bladder leiomyosarcoma and the lack of standardized treatment guidelines, management remains controversial and is often extrapolated from treatment approaches for soft tissue sarcomas at other sites. In the past decade, fewer than 20 cases have been presented in the literature (Supplementary Table S1). The largest case series to date by Dayron et al. which studied 183 cases of bladder leiomyosarcomas, reported that radical cystectomy was the most commonly employed treatment modality, performed in 52% of patients [11]. Radical cystectomy is generally considered the gold standard for localized disease [17]. Their study highlighted that complete surgical resection and the absence of distant metastases at diagnosis were associated with improved overall survival, with a reported median survival of 46 months [11]. These findings underscore the importance of aggressive initial management in achieving favorable outcomes, particularly in patients without evidence of metastasis at the time of surgery. However, our case deviates from this cohort in several aspects. Although the tumor was graded as moderately differentiated (G2), it exhibited a high mitotic index and extensive necrosis, features more typical of poorly differentiated leiomyosarcomas. Additionally, desmin negativity on immunohistochemistry, along with extensive local invasion into adjacent organs (ureter, vagina, and sigmoid colon), and early systemic metastases within a year after surgery, represent uncommon findings not detailed in the Rodriguez et al. series. In our case, radical tumor resection required cystectomy and resection of adherent bowel segments due to extensive local invasion. Despite radical tumor resection and the absence of metastases at the time of diagnosis, the development of widespread distant disease one year after surgery indicates an unfavorable prognosis and limited long-term survival. A similar case described initial management with TURBT followed by radical cystectomy, after which no metastatic disease was detected at the six-month postoperative follow-up [18].

Although no standardized regimen exists, chemotherapy is frequently employed in metastatic or high-grade cases. Beyond tumor-specific characteristics, recent research has also underscored the prognostic relevance of host-related factors such as systemic inflammation and smoking status. Ferro et al. demonstrated that bladder cancer patients with elevated inflammatory markers—including NLR, PLR, and LMR—particularly among smokers, exhibited a significantly increased risk of disease progression despite BCG therapy [19]. These findings highlight the broader impact of the tumor microenvironment and systemic immune status on therapeutic outcomes in urothelial malignancies. The combination of doxorubicin and dacarbazine has shown efficacy in soft tissue sarcomas [20]. Doxorubicin, an anthracycline, induces DNA damage via intercalation and topoisomerase II inhibition, while dacarbazine functions as an alkylating agent, causing apoptosis through DNA methylation [21]. Our patient initially responded to chemotherapy, as demonstrated by temporary metastatic regression after six cycles; however, disease progression resumed within months, requiring initiation of palliative systemic therapy.

The published literature suggests a median survival of approximately thirty months following surgery, with survival rarely exceeding six months in untreated patients [11,22,23,24,25,26,27,28,29,30,31,32,33,34,35,36,37,38,39,40]. Although aggressive multimodal therapy was pursued in our case, the recurrence of metastases and subsequent palliative management illustrate the poor prognosis associated with advanced bladder leiomyosarcoma.

Given the rarity of this tumor, treatment recommendations are largely extrapolated from data on soft tissue sarcomas at other sites. This case contributes to the evolving evidence base supporting individualized, multimodal treatment strategies for bladder leiomyosarcoma.

## 4. Conclusions

Bladder leiomyosarcoma poses significant diagnostic and therapeutic challenges due to its rarity, aggressive nature, and lack of standardized management protocols. Despite initial non-metastatic presentation and radical surgery, disease progression required palliative care, reflecting the tumor’s aggressive course and limited long-term outcomes. It highlights the importance of tailored approaches and vigilant postoperative monitoring. Collaborative efforts and larger clinical datasets are urgently needed to inform evidence-based guidelines for this rare malignancy.

## Data Availability

Due to the case-report nature of this methodology, the data presented in this study are available on request from the corresponding author. No new data were created or analyzed in this study.

## Supplementary Materials

The following supporting information can be downloaded at: https://www.mdpi.com/article/10.3390/jcm14175999/s1, Table S1: Summary of Prior Case Reports of Primary Bladder Leiomyosarcoma published between 2015 and 2025. References [22,23,24,25,26,27,28,29,30,31,32,33,34,35,36,37,38,39,40] are cited in Supplementary Materials.
