# Supplementary material for: Rare and Aggressive Disease: Urinary Bladder Leiomyosarcoma
Source: J Clin Med. 2025 Aug 25;14(17):5999. doi: 10.3390/jcm14175999 (PMC12429293; doi:10.3390/jcm14175999)
Supplement: Supplementary file 1 [file jcm-14-05999-s001.zip › jcm-3754977-supplementary.pdf]

## Supplementary Table S1. Summary of Prior Case Reports of Primary Bladder Leiomyosarcoma published between 2015 and 2025

| Study (Author, Year) | No. of patients | Age | Sex | Presentation | Treatment | Histology | Outcome / Follow-Up |
|---|---|---|---|---|---|---|---|
| Uhlman MA et al., 2018 | 1 | 26 | F | Hematuria | Bladder-neck-sparing partial cystectomy and "W"-pouch neobladder; negative margins confirmed on frozen section | Low-grade leiomyosarcoma | Bladder-neck-sparing partial cystectomy and "W"-pouch neobladder; negative margins confirmed on frozen section |
| Vias P et al., 2021 | 1 | 45 | F | Dysuria, hematuria | TURBT, surgical resection, systemic chemotherapy, pelvic radiation | High-grade, aggressive leiomyosarcoma | Recurrence in intestinal mucosa and peritoneum; overall survival ~1.7 years despite aggressive therapy |
| Hoshina H et al., 2023 | 1 | 37 | F | 17 weeks pregnant, history of childhood retinoblastoma, gross hematuria; cystoscopy revealed a 40 mm papillary lesion on the lateral bladder wall | TURBT at 25 weeks of gestation; after C-section at 31 weeks, definitive partial cystectomy with no residual tumor | Leiomyosarcoma with loss of RB1 expression | No residual disease on specimen; maternal and fetal outcomes favorable |
| Anakievski D et al., 2019 | 1 | Not specified | M | Gross hematuria, dysuria, pollakiuria in a male patient; tumor detected via imaging | Laparoscopic radical cystoprostatectomy with totally intracorporeal orthotopic ileal neobladder diversion | High-grade leiomyosarcoma | No evidence of local or distant recurrence at 12-month follow-up |
| Tan Z et al., 2021 | 1 | 20 | F | 2.5 × 3.0 cm lobulated mass on left lateral bladder wall | Initial TURBT, repeat TURBT at 30 days; followed by adjuvant chemotherapy | LMS has been subdivided into low- and high-grade based on nuclear atypia, | Patient doing well at 12-month follow-up with no recurrence reported |

| | | | | | | | |
|---|---|---|---|---|---|---|---|
| | | | | discovered in a 20-year-old female with hematuria. | (ifosfamide + doxorubicin). | mitotic activity, and tumor necrosis. | |
| Hart AA et al., 2022 | 1 | 56 | M | Gross hematuria, dysuria. CT revealed a 9.2 cm bladder mass invading the prostate, bilateral hydronephrosis; no nodal or distant metastases. | Radical cystoprostatectomy with pelvic lymph node dissection and ileal conduit urinary diversion | High-grade (25 mitoses/10 HPF, 10% necrosis), grade 3 tumor with lymphovascular invasion. | Negative margins, negative nodes; no recurrence at 9-month post-op imaging |
| Doddamani SC et al., 2015 | 1 | 30 | M | painless hematuria with clots of 2 months' duration. At 3 years of age, he was diagnosed to have retinoblastoma in both eyes and was treated with 18 cycles of radiation | He was not willing to undergo radical surgery and was referred to the oncologist for further management and subsequently lost to follow-up | a high-grade sarcoma with only spindle cells and no epithelial cells | Patient lost to follow-up |
| Fiorentino V et al., 2019 | 1 | 86 | M | The patient was referred to the institution to undergo endoscopic low-urinary-tract re-evaluation 2 months after the detection of a “low-grade urothelial neoplasia” in | Transurethral resection of the bladder tumor (TURBT) with complete local excision of the 3.5 cm mass on the left bladder wall | well-differentiated leiomyosarcoma (T1N0M0; G3) with clear surgical margins | At follow-up, including regular cystoscopy and imaging over 18 months, the patient remained free of local recurrence or distant metastasis. |

| | | | | | | | |
|---|---|---|---|---|---|---|---|
| | | | | urinary cytology. | | | |
| Patnayak, R et al., 2015 | 1 | 46 | M | dysuria, burning micturition, increased frequency and urgency since 6 months. Hematuria for last 2-3 days without any history of obstructive symptoms | Radical cystectomy with lymphadenectomy | interlacing fascicles of pleomorphic spindle cells, high mitotic activity, and necrotic areas | The patient was disease-free at 3 years post-surgery |
| Ribeiro JG et al., 2016 | 1 | 31 | F | progressive symptoms of vesical irritability associated with macroscopic hematuria, with a history of frequent urinary tract infections | Complete resection along with free margins | a low grade leiomyosarcoma | The postoperative period was uneventful and no complementary treatment was given to the patient. During 12 months of follow-up, the patient showed no signs of tumour recurrence |
| Zeng Z et al., 2023 | 1 | 44 | M | Dysuria for 2 weeks | A robot-assisted laparoscopic enucleation of the bladder tumor. Underwent 5 cycles of adjuvant chemotherapy after surgery | low-grade LMS-UB | At 19months postoperative follow-up, the patient had no symptoms, recurrence, or distant metastasis |
| Ishizaki A et al., 2020 | 1 | 65 | F | metastatic pancreatic leiomyosarcoma originating from a primary urinary bladder tumor | Systemic chemotherapy with doxorubicin at 75 mg/m² administered after the diagnosis | MIB-1 labeling index of 20% supported the diagnosis of high-grade leiomyosarcoma | Minimally invasive diagnosis via EUS-FNB enabled timely systemic treatment |
| Sato R et al., 2018 | 1 | 49 | F | A 12-month history of frequent | Cystoscopy and TURBT | Mitotic figures were observed | Disease-free 18 months after surgery |

| | | | | | | | |
|---|---|---|---|---|---|---|---|
| | | | | urinary tract infections presented with progressive symptoms of dysuria. | | frequently (3/HPF in hot spot). | |
| Anastasiou A et al., 2018 | 1 | 43 | M | Painless macroscopic hematuria | Transurethral resection of the tumor and aggressive conservative management. | N/A | The patient a year after the diagnosis is in perfect condition without signs of a recurrence or metastases. |
| Cuypers A et al., 2025 | 1 | 57 | F | Gross haematuria and an abnormality in the urinary bladder on an abdominal ultrasound. | Transurethral en-bloc resection of bladder mass. After recovery, a robot-assisted partial cystectomy with a margin of more than 1 cm from the scar was performed. | Low-grade leiomyosarcoma | The patient recovered well from the procedure and currently remains under surveillance. |
| Fakhoury M et al., 2016 | 1 | 77 | F | Microscopic hematuria, complaints of dysuria and pelvic pain upon urination. | Transurethral resection of the bladder tumor and subsequently underwent radical anterior pelvic exenteration along with the creation of an ileal conduit. | Up to 14 mitoses per 10 high-power fields are identified, where several areas of tumor necrosis are noted. | The hospital course was uneventful and she was discharged on post-operative day 8 and is currently without evidence of recurrence. |
| Li Y et al., 2022 | 1 | 70 | M | Macroscopic hematuria accompanied by dysuria, and a history of urinary bladder carcinoma 3 years prior. | Laparoscopic radical cystectomy. | FNCLCC Grade III. | With 45 months of follow-up, we have not found any sign of recurrence to date. |

| | | | | | | | |
|---|---|---|---|---|---|---|---|
| Sevilla CR et al., 2018 | 1 | 26 | M | History of neuroblastoma of the right eye treated with cyclophosphamide and esphenoidal meningioma. | Radical cystectomy. | N/A | 8th year of follow-up after the radical surgery, the patient is free of bladder disease. |
| Ohan H at al., 2020 | 1 | 24 | M | N/A | N/A | N/A | N/A |
| Boutaggount et al., 2022 | 1 | 48 | M | Macroscopic hematuria and symptoms of bladder irritation with severe anemia (hemoglobin of 4 g/dL). | TURB followed by a radical cystectomy with pelvic nodes dissection and Briker type derivation ensuring R0 surgery. | FNCLCC Grade II leiomyosarcoma. | The patient remained free of recurrence 11 months after his treatment. |

M – male

F – female

FNCLCC - The Federation Nationale des Centres de Lutte Contre le Cancer - grading system used by doctors to evaluate soft tissue sarcomas.

TURBT – transurethral resection of bladder tumor

N/A – not available

Mo – months

3/HPF- 3 mitotic figures per High Power Field.
